# Relevance of Partitioning DLCO to Detect Pulmonary Hypertension in Systemic Sclerosis

**DOI:** 10.1371/journal.pone.0078001

**Published:** 2013-10-18

**Authors:** Nadia Sivova, David Launay, Lidwine Wémeau-Stervinou, Pascal De Groote, Martine Remy-Jardin, Guillaume Denis, Marc Lambert, Nicolas Lamblin, Sandrine Morell-Dubois, Marie Fertin, Guillaume Lefevre, Vincent Sobanski, Olivier Le Rouzic, Pierre-Yves Hatron, Benoit Wallaert, Eric Hachulla, Thierry Perez

**Affiliations:** 1 Service de Médecine Interne, Centre de Référence des Maladies Autoimmunes et Systémiques Rares (Sclérodermie), Centre de Compétence de l’Hypertension Artérielle Pulmonaire Sévère, Université Lille Nord de France, Hôpital Claude-Huriez, CHRU Lille, Lille, France; 2 Laboratoire d’Immunologie EA2686, Université Lille Nord de France, Faculté de Médecine, Lille, France; 3 Clinique des Maladies Respiratoires, Centre de Compétence des Maladies Pulmonaires Rares, Centre de Compétence de l’Hypertension Artérielle Pulmonaire Sévère, Hôpital Calmette, Université Lille Nord de France, CHRU Lille, Lille, France; 4 Service de Cardiologie, Centre de Compétence de l’Hypertension Artérielle Pulmonaire Sévère, Université Lille Nord de France, Hôpital Cardiologique, CHRU Lille, Lille, France; 5 Service de Radiologie Thoracique, Université Lille Nord de France, Hôpital Calmette, CHRU Lille, Lille, France; 6 Service d’Explorations Fonctionnelles Respiratoires, Université Lille Nord de France, Hôpital Calmette, CHRU Lille, Lille, France; Keio University School of Medicine, Japan

## Abstract

We investigated whether partitioning DLCO into membrane conductance for CO (DmCO) and pulmonary capillary blood volume (Vcap) was helpful in suspecting precapillary pulmonary (arterial) hypertension (P(A)H) in systemic sclerosis (SSc) patients with or without interstitial lung disease (ILD). We included 63 SSc patients with isolated PAH (n=6), isolated ILD (n=19), association of both (n=12) or without PAH and ILD (n=26). Partitioning of DLCO was performed by the combined DLNO/DLCO method. DLCO, DmCO and Vcap were equally reduced in patients with isolated PAH and patients with isolated ILD but Vcap/alveolar volume (VA) ratio was significantly lower in the isolated PAH group. In patients without ILD, DLCO, DmCO, Vcap and Vcap/VA ratio were reduced in patients with isolated PAH when compared to patients without PAH and both Vcap/VA and DLCO had the highest AUC to detect PAH. In patients with ILD, Vcap had the highest AUC and performed better than DLCO to detect PH in this subgroup. In conclusion, Vcap/VA was lower in PAH than in ILD in SSC whereas DLCO was not different. Vcap/VA ratio and DLCO had similar high performance to detect PAH in patients without ILD. Vcap had better AUC than DLCO, DmCO and FVC/DLCO ratio to detect PH in SSC patients with ILD. These results suggest that partitioning of DLCO might be of interest to detect P(A)H in SSC patients with or without ILD.

## Introduction

Precapillary pulmonary hypertension (PH) is frequent in systemic sclerosis (SSc) and is one of the leading causes of deaths in this disease [1,2]. In SSc, precapillary PH can be either isolated (pulmonary arterial hypertension or PAH) or be associated with ILD [3,4,5,6,7,8]. The prevalence of PH is high (5-10%) and despite recent advances in the treatment strategy and armamentarium, its prognosis remains poor with a median survival of 3 years [6,9,10]. Therefore, in SSc, an annual screening is recommended by echocardiography and pulmonary functional tests (PFT) with determination of the diffusion capacity of the lungs for carbon monoxide (DLCO) [8,11]. Modification of DLCO can be explained by changes in gas exchange area, alteration of the alveolar capillary membrane, ventilation/perfusion relationship in the lung and pulmonary capillaries involvement [12]. Therefore, DLCO is reduced in patients with PH [13]. Some authors also suggested that a forced vital capacity (FVC)/DLCO ratio > 1.6 (percentage predicted) was useful in the diagnosis of pulmonary vasculopathy in SSc patients with or without ILD [14]. However, the exact role and the sensitivity/specificity of a low DLCO to predict the existence of PH are still poorly known. Moreover, another unsolved problem is the interpretation of a low DLCO in a patient with ILD, as ILD itself is a cause of decrease in DLCO.

DLCO can be partitioned into two transfer components or conductances arranged in series, namely membrane conductance for CO (DmCO), which reflects the diffusion properties of the alveolar capillary membrane, and CO loading on hemoglobin (Hb), which is the product of the CO-Hb chemical reaction rate (θCO) and the mass of Hb in the alveolar capillary blood volume (Vcap) [15]. These 2 conductances are related by the equation proposed by Roughton and Forster: 1/DLCO = 1/DmCO + 1/θCOxVcap [15]. A decreased DmCO is interpreted as a ‘thickening’ of the alveolo-capillary membrane or as a decrease in lung area, and a decreased Vcap is viewed as a lowered blood volume in the ventilated alveoli [16]. DmCO and Vcap can be determined by two methods: duplicate measurements of DLCO with high and low O2 concentrations [15], or the DLNO/DLCO technique more recently described by Guenard et al [17]. Data are scarce and limited on the relevance of DmCO and Vcap in assessing lung involvement in SSc and especially the presence of PAH. One study suggested that DmCO/Vcap ratio was interesting in the diagnosis of ILD in SSc but patients with PH were excluded [18]. Another study found no role in partitioning DLCO for the diagnosis of PH in SSc [19]. However, in the latter study, although results were adjusted for total fibrosis score, patients with ILD were mixed in the PH group and the control group [19].

The aim of our study was to assess the role of partitioning of DLCO in the diagnosis of PH both in patients with and without a coexisting ILD 

## Methods

We retrospectively included all consecutive patients attending the National Reference Center for scleroderma (Lille) between January 2006 and December 2010 if 1. They fulfilled the American College of Rheumatology (ACR) criteria for SSc and/or the LeRoy’s classification system (limited cutaneous or diffuse cutaneous SSc) [20,21]. 2. They had available pulmonary function tests with DLCO/DLNO and echocardiography data. Patients were excluded if 1. They had another pulmonary disease than ILD (including obstructive disease), left heart failure (defined by a left ventricle ejection fraction < 55%) or a history of pulmonary embolism; or 2. They had no available right heart catheterization while having a tricuspid regurgitation velocity > 2.8 m/s or additional echocardiographic variables suggesting the presence of PH. 

This study was authorized by the French Competent Authority dealing with Research on Human Beings, namely the French Ministry of Reasearch and was approved by the French pulmonology society ("*Société de Pneumologie de Langue Française* institutional review board (CEPRO 2012 009) and compliant with requirements of the “Commission Nationale de l’Informatique et des Libertés”. To issue such authorization, the Ministry of Reasearch and the French pulmonology society sought the advice of an institutional review board, namely the “Comité de Protection des Personnes” which voted positively on the quality of information provided to patients and approved oral consent. All patients provided informed oral consent to participate because written consent is not mandatory in retrospective and officially approved studies according to the French laws. Oral consent was documented in the medical files of each patient.

### Pulmonary function tests

Resting pulmonary function tests included measurement of lung volumes by plethysmography and single breath DLCO (Jaeger-Masterlab ® plethysmograph) was performed in all patients. The reference values used were those of the Official Statement of the European Respiratory Society [22,23]. Percentages of predicted for DLCO and KCO (DLCO/alveolar volume ratio) are abbreviated as DLCO% and KCO% and were corrected for measured haemoglobin according to ATS/ERS guidelines [22,23]. Non-encouraged 6-minute walk test (6MWT) was performed following ATS recommendations [24]. New York Heart Association (NYHA) functional class was assessed.

### DLNO and DLCO

In all patients, diffusing capacity of the lung using the transfer gas nitric oxide (DLNO) and DLCO were measured simultaneously during a single breath manœuvre using an automatic apparatus (Medisoft Dinant®, Belgium) as described by Aguilaniu et al [25]. Briefly, the measurements of DLNO/DLCO were accepted if two successive measurements of DLNO and DLCO gave results within 10%, otherwise a third measurement was performed. Values of DmCO and Vcap were calculated according to the model of Guénard et al. [17] 1/θNO was assumed negligible, therefore DLNO=DmNO. DmCO was calculated as DmNO/a where a=1.97 following Graham’s law. To calculate Vcap, θCO reference from Roughton and Forster was chosen [15]. Vcap was corrected for haemoglobin concentration. For male patients, measured Vcap was multiplied by 14.6/measured haemoglobin to obtain the corrected Vcap. For female patients, measured Vcap was multiplied by 13.4/measured haemoglobin to obtain the corrected Vcap. We report here only the corrected Vcap. Reference values for DmCO and Vcap used were those established by Aguilaniu et al. [25]. DmCO% and Vcap% are the % of predicted values for DmCO and Vcap respectively. Vcap/VA ratio is the ratio of Vcap in mL and alveolar volume in L.

### Definition of interstitial lung disease and pulmonary hypertension

All patients had underwent a HRCT of the chest within 36 months of lung function assessment. ILD was defined by the presence of at least one usual sign of SSc-associated ILD, i.e. subpleural pure ground glass opacities and/or interstitial reticular pattern with or without traction bronchiectasis, and/or honeycomb cysts [26,27]. The extension of ILD was graded as limited or extensive on HRCT following the recently published Goh’s staging system [27]. We collected in the patient file the presence or absence of a known precapillary PH at the time of lung function assessment. Precapillary PH was defined in each cases as mean pulmonary arterial pressure (mPAP) ≥25 mm Hg at rest and pulmonary artery wedge pressure (PAWP) ≤15 mm Hg measured during a right heart catheterisation at rest. All patients, with or without a known PH, underwent a transthoracic echocardiography within 3 months of lung function assessment. According to recent guidelines [8], in patients without a known PH, PH was considered as unlikely when tricuspid regurgitation velocity was ≤ 2.8 m/s and no additional echocardiographic variables suggestive of PH were present. 

We defined 4 groups: 1. Patients without ILD on the last HRCT and unlikely to have PH as defined above (noILD noPH group). 2. Patients with isolated precapillary PAH on right heart catheterization without any ILD manifestations on the last HRCT (PAH group) 3. Patients with ILD on the last HRCT, whatever the extension of ILD (i.e. limited or extensive according to Goh’s staging system [27]), and unlikely to have PH as defined above (ILD group) 4. Patients both with ILD of any extension on the last HRCT (i.e. limited or extensive according to Goh’s staging system [27]) and precapillary PAH on right heart catheterization (ILD-PH group).

### Statistical analysis

Categorical data were described by frequency and percentages; continuous data were summarised by their median and range. Data were analysed using the SPSS software. Comparisons between groups were performed using Kruskal-Wallis or Wilcoxon’s test for continuous variables and chi square test or Fisher’s exact test for categorical variables. Correlations were analysed using Spearman’s rank correlation test. Receiver operating characteristic (ROC) curves were constructed to illustrate the power of different parameters to detect PH or PAH over an entire range of cutoff values. The overall diagnostic accuracies of the tests were estimated using the area under the ROC curve. Figures were built using Prism5 software (San Diego, California, USA).

## Results

### Characteristics of the population

63 patients (51 females; median age: 55 (25-84) yrs) were enrolled in this retrospective study: 26 fulfilled the noILDnoPH group criteria, 6 the PAH group, 19 the ILD group and 12 the ILD-PH group criteria. No patients had evidence of combined pulmonary fibrosis-emphysema syndrome. In the ILD group, 9 patients had an extensive ILD and 10 a limited ILD. In the ILD-PH group, 9 had an extensive ILD and 3 a limited ILD according to Goh’s staging system [27]. In the noILDnoPH group, 19 patients had no immunosuppressive treatments at inclusion while 4 received corticosteroids, 1 corticosteroids and mycophenolate mofetil and 2 methotrexate. In the PAH group, no patients received immunosupressants, 3 were treated by bosentan, 1 by epoprostenol and bosentan and 1 by trepostinil associated with bosentan and sildenafil. One PAH patient was not treated for PAH as PAH was considered as mild. In the ILD group, 10 patients received corticosteroids and mycophenolate mofetil, 2 corticosteroids alone, 1 corticosteroids and azathioprine, 1 corticosteroids and methotrexate and 1 cyclophosphamide while 4 patients were not treated. In the ILD-PH group, immunosuppressive therapy was given in 9 patients (4 corticosteroids and cyclophosphamide, 2 corticosteroids alone, 2 corticosteroids and mycophenolate mofetil and 1 corticosteroids and azathioprine). Nine ILD-PH patients received PH treatment (4 bosentan, 1 bosentan with sildenafil, 1 epoprostenol with bosentan, 1 trepostinil with bosentan and 2 trepostinil with bosentan and sildenafil). Characteristics of the whole population and the 4 groups are summarized in Table 1. 

**Table 1:** Baseline characteristics of the patients.

	Whole population (n=63)	noILDnoPH group (n=26)	PAH group (n=6)	ILD group (n=19)	ILD-PH group (n=12)	P between groups
Age (yrs)	55 (25-84)	52.5 (32-77)	68 (45-78)	52 (25-82)	66.5 (45-84)	<0.05
Females/males (n (%)	51 (81)/12 (19)	24 (92)/2 (8)	6 (100)/0 (0)	12 (63)/7 (34)	9 (75)/3 (25)	NS
Height (cm)	1.63 (1.50-1.90)	1.64 (1.50-1.90)	1.60 (1.50-1.70)	1.65 (1.50-1.81)	1.59 (1.50-1.74)	NS
Weight (kg)	65 (45-112)	63.5 (45-104)	57.5 (49-77)	70 (49-105)	66.5 (47-112)	NS
Ever smoked/never smoked	15/48	8/18	1/5	3/16	3/9	NS
Limited/diffuse SSc (n (%)	50 (79)/13 (21)	23 (88)/3 (12)	5 (83)/1 (17)	12 (63)/7 (37)	10 (83)/2 (17)	NS
Duration of SSc (yrs)	8 (1-27)	9 (1-19)	10 (4-24)	5 (1-23)	11 (4-27)	NS
NYHA functional class I/II/III/IV (n (%)	18 (28)/25 (40)/15 (24)/5 (8)	14 (54)/10 (38)/2 (8)/0 (0)	1 (17)/2 (33)/3 (50)/0 (0)	3 (16)/12 (63)/3 (16)/1 (5)	0 (0)/1 (8)/7 (58)/4 (33)	<0.05
6-min walk test (m)	403 (200-708)	417 (300-708)	427 (290-534)	401 (200-570)	315 (222-480)	<0.05
Systolic PAP (mmHg)	32 (15-106)	27 (15-37)	44 (37-95)	32 (17-40)	56 (32-106)	<0.05
tricuspid regurgitation velocity (ms^-1^)	2.59 (1.58-5.02)	2.34 (1.58-2.78)	3.12 (2.82-4.74)	2.59 (1.73-2.73)	3.53 (2.60-5.02)	<0.05
Mean PAP (mmHg) on right heart catheterization	NA	NA	26 (25-60)	NA	33 (25-53)	NA
FVC L/% predicted	2.62 (1.44-4.86)/93 (42-139)	3.24 (1.97-4.77)/103 (42-139)	2.29 (1.65-3.46)/91 (77-99)	2.61 (1.53-4.86)/89 (46-124)	1.92 (1.44-3.20)/78 (52-112)	<0.05
TLC L/% predicted	4.70 (2.15-8.09)/94 (56-127)	5.29 (4.04-7.71)/105 (79-126)	4.27 (3.74-6.22)/90 (82-110)	4.30 (3.09-8.09)/80 (65-123)	3.59 (2.15-5.91)/73 (57-100)	<0.05
Hemoglobin, g/dL	13 (9.1-16)	13 (10-14.6)	13.4 (12.1-13.7)	13.5 (10.4-15.4)	12.8 (9.1-16)	NS
DLCO mL/min/mmHg/%predicted	13.44 (2.35-29.00)/58 (11-111)	19.40 (7.11-26.30)/78 (37-111)	10.71 (5.57-15.12)/44 (27-56)	12.36 (7.17-29.00)/52 (34-87)	6.49 (2.35-11.00)/29 (11-63)	<0.05
KCO mL/min/mmHg/%predicted	3.64 (0.39-5.41)/77 (8-114)	4.18 (0.39-5.41)/85 (8-114)	2.74 (1.65-4.68)/59 (34-110)	3.60 (2.26-4.96)/76 (50-102)	2.02 (0.72-4.19)/47 (19-98)	<0.05
Vcap mL/% predicted	35.9 (5.7-90.8)/63 (8-145)	48.9 (18.3-82.1)/67 (29-145)	26.0 (22.1-45.1)/60 (34-89)	36.3 (20.4-90.8)/53 (27-142)	16.6 (5.7-24.6)/33 (8-139)	<0.05
DmCO mL/min/mmHg/%predicted	29.2 (10.6-69.7)/49 (21-101)	44.9 (20.2-62.2)/66 (35-101)	23.6 (13.6-35.9)/40 (32-57)	25.5 (13.4-69.7)/38 (22-85)	16.3 (10.6-30.5)/31 (21-51)	<0.05
Vcap/VA ratio	10.3 (2.5-22.7)	11.6 (5.8-16.5)	7.6 (6.6-8.3)	10.9 (4.6-22.7)	5.2 (2.5-13.2)	<0.05
DmCO/Vcap ratio	0.89 (0.26-2.04)	0.91 (0.44-1.71)	0.77 (0.61-1.34)	0.80 (0.26-1.53)	1.13 (0.75-2.04)	<0.05
DLNO/DLCO	4.44 (2.60-12.00)	4.41 (2.60-7.08)	4.29 (3.59-6.57)	4.07 (3.34-5.19)	5.69 (3.16-11.97)	NS
FVC/DLCO%	1.60 (0.54-6.20)	1.37 (0.54-2.46)	2.00 (1.75-3.25)	1.60 (1.13-2.85)	2.75 (1.60-6.20)	<0.05

SSc: systemic sclerosis ; PAP: pulmonary arterial pressure ; FVC: forced vital capacity ; TLC: total lung capacity ; DLCO: diffusion capacity of the lungs for carbon monoxide ; Vcap: alveolar capillary blood volum: membrane conductance for CO DmCO ; DLNO: diffusion capacity of the lungs for nitric oxide; VA: alveolar volume

### Comparison of DLCO, Vcap and DmCO between patients with isolated PAH and patients with isolated ILD

**Figure 1:**
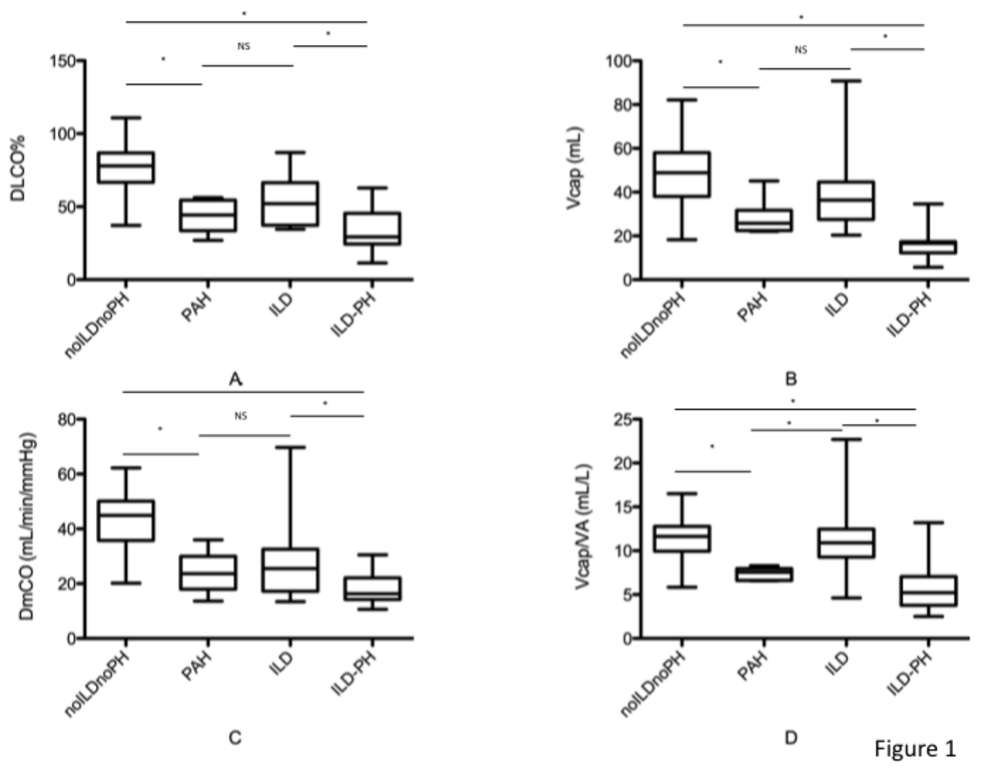
Comparison of DLCO% of predicted (panel A), Vcap (panel B), DmCO (panel C) and Vcap/VA ratio (panel D) between SSc patients with PAH, ILD, both ILD and PH (ILD-PH group) and no complications (noILDnoPH group). NS: non significant; *: p<0.05.

There was no difference in DLCO, DLCO%, KCO, KCO%, FVC%/DLCO%, DmCO, DmCO%, Vcap, Vcap%, DmCO/Vcap and DLNO/DLCO ratio between patients in the isolated PAH group and patients in the isolated ILD group (Table 1 and Figure 1A, 1B and 1C). Conversely, Vcap/VA ratio was significantly reduced in the PAH group when compared to the ILD group (7.6 (6.6-8.3) vs 10.9 (4.6-22.7 mL/L, p<0.05) (Figure 1D). Results remained unchanged after adjusting for smoking status. ROC analysis revealed that a Vcap/VA ratio < 8.6 mL/L had a sensitivity of 100%, a specificity of 89 % and a positive likelihood ratio of 8 to identify PAH patients. Positive and negative predictive values were 75% and 100% respectively.

### Role of DLCO and its partitioning in the diagnosis of PAH in a SSc patient without ILD

In this section, we focus on SSc patients without ILD on HRCT scan either with (PAH group) or without PAH (noILDnoPH group). When compared to the noILDnoPH group, patients with PAH had a significantly lower DLCO, DLCO%, KCO, KCO%, Vcap, DmCO, DmCO% and Vcap/VA ratio as well as a higher FVC/DLCO ratio (Table 1 and Figure 1A, 1B, 1C and 1D). Conversely, there was no difference in Vcap%, DmCO/Vcap and DLNO/DLCO ratio. Results remained unchanged after adjusting for smoking status

**Figure 2:**
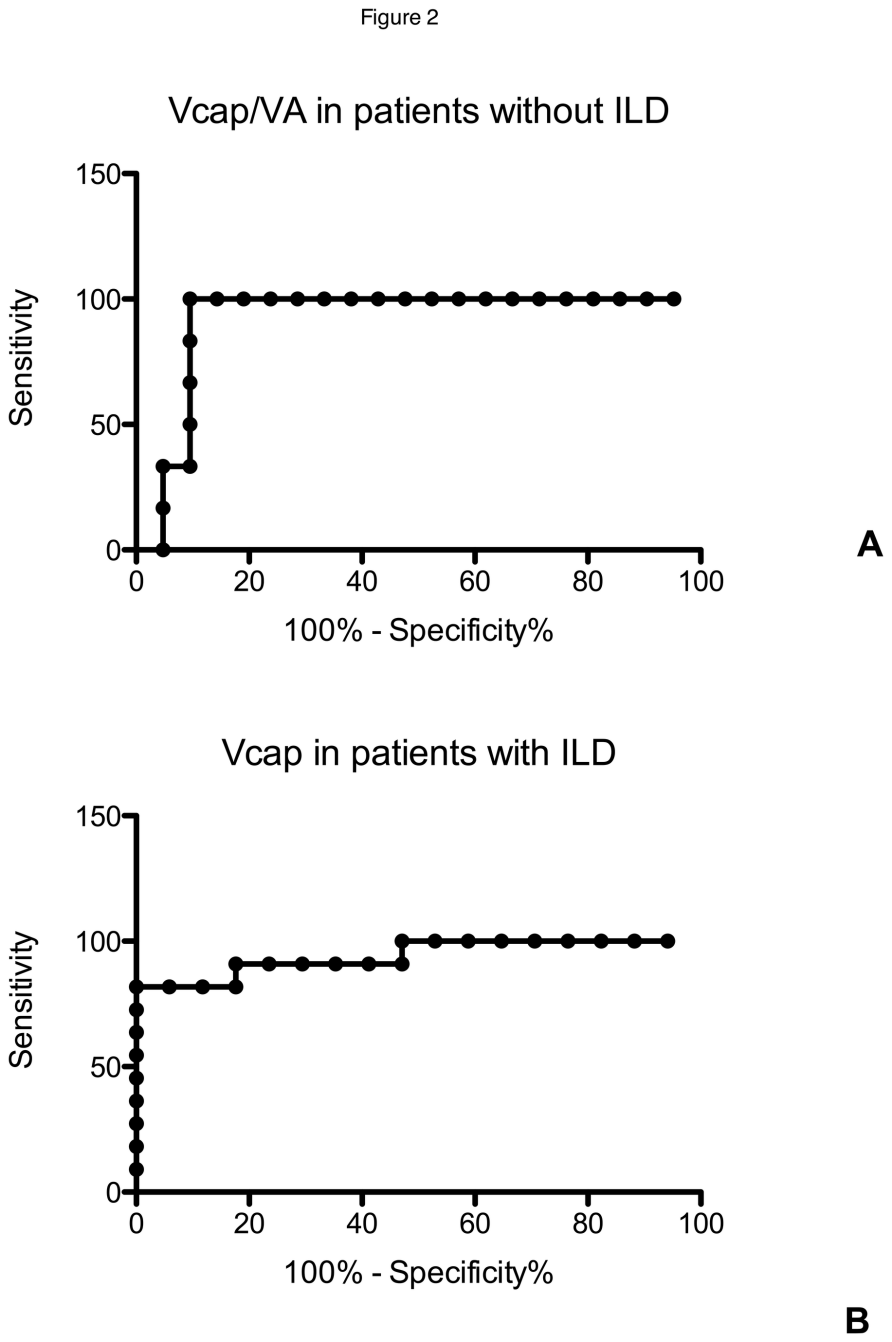
ROC curve analysis of Vcap/VA ratio for the detection of PAH in patients without ILD (panel A) and Vcap for the diagnosis of PH in patients with ILD (panel B).

By ROC analysis, the highest AUC for the diagnosis of PAH were observed for Vcap/VA (0.93) (Figure 2A), DLCO% (0.93) and FVC/DLCO (0.93). DmCO, Vcap, and KCO%, had an AUC of 0.92, 0.90, and 0.78 respectively. A Vcap/VA <8.6 mL/L had a sensitivity of 100%, a specificity of 90% and positive likelihood ratio of 10.5 to detect PAH in this subgroup of SSc patients without ILD. Positive and negative predictive values were 75% and 100% respectively. For a sensitivity of 100%, DLCO%<57% had a specificity of 88% and FVC/DLCO>1.6 a specificity of 77%. Tricuspid regurgitation velocity > 2.8 m/s had a sensitivity of 66%.

### Role of DLCO and its partitioning in the diagnosis of PH in a patient with ILD

In this section, we focus on SSc patients with ILD on HRCT scan either with (ILD-PH group) or without PH (isolated ILD group). When compared to patients with isolated ILD, patients in the ILD-PH group had a significantly lower DLCO, DLCO%, KCO, KCO%, Vcap, DmCO, Vcap/VA ratio and a higher DmCO/Vcap, FVC%/DLCO% and DLNO/DLCO ratio (Table 1 and Figure 1A, 1B, 1C and 1D). Conversely, Vcap% and DmCO% were not significantly different. Results remained unchanged after adjusting for smoking status.

By ROC analysis, the highest AUC for the diagnosis of PH was observed for Vcap (0.94) (Figure 2B). Vcap/VA, DLCO%, FVC%/DLCO%, DmCO and DLNO/DLCO had a lower AUC of 0.87, 0.86, 0.82, 0.79 and 0.76 respectively. To achieve a specificity of 100% for the detection of PH in these SSc patients with ILD, Vcap<19 mL had a sensitivity of 83%, DLCO%<34% a sensitivity of 72%, KCO%<50% a sensitivity of 42% and FVC%/DLCO% > 2.94 a sensitivity of 50%. FVC%/DLCO%>1.6 had a sensitivity of 100% but a low specificity of 50%. Vcap<19 mL had therefore a sensitivity, a specificity, positive and negative predictive values of 83%, 100%, 100% and 90% respectively. Tricuspid regurgitation velocity > 2.8 m/s had a sensitivity of 66%.

Finally, when we pooled on one side patients with right heart catheterization-proven PH either with or without associated ILD (i.e. PAH and ILD-PH groups, n=18) and on the other side patients without PH either with or without ILD (i.e. noILDnoPH and ILD groups, n=45), we found that Vcap had the best AUC of 0.91 to detect P(A)H in SSc patient whatever the presence of ILD whereas Vcap/VA, DLCO%, FVC/DLCO or DmCO had lower AUC (0.89, 0.89, 0.88 and 0.87 respectively). Vcap <19 mL had a specificity of 100% and a sensitivity of 53% for the presence of P(A)H. DLCO%<33% had the same sensitivity for a specificity of 100%. Of note, Vcap/VA ratio and Vcap were significantly lower in the 18 patients with PAH and ILD-PH than in the 45 patients with noILDnoPH and ILD and these results persisted after correction for age at evaluation and smoking status (data not shown).

### Correlation between DmCO, Vcap, Vcap/VA, DLCO and FVC/DLCO ratio with systolic PAP in the whole population

**Figure 3:**
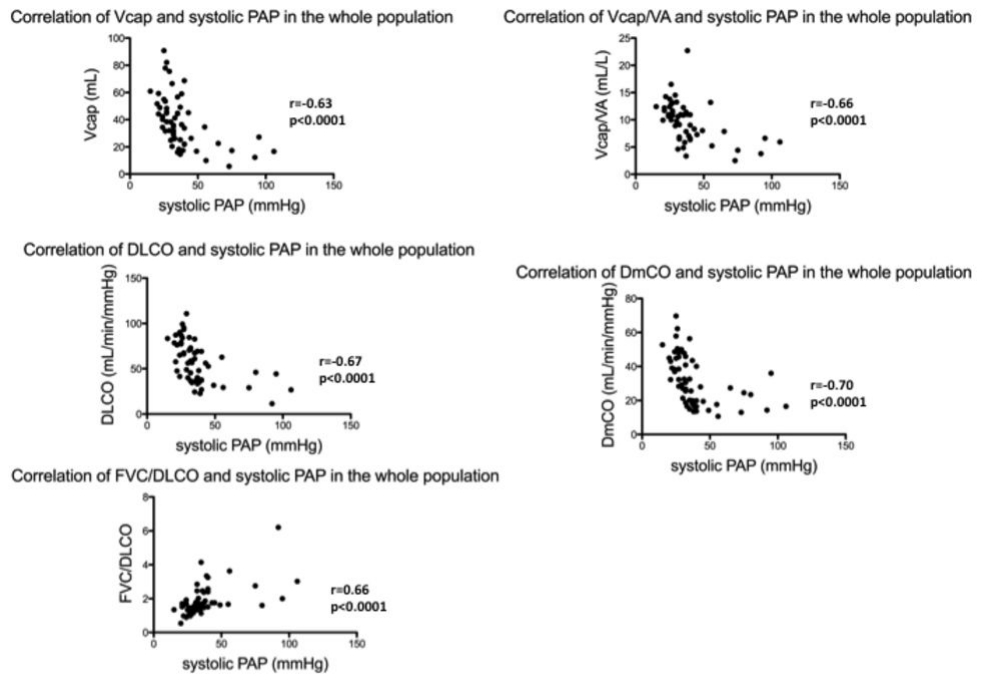
Correlations between DLCO, Vcap, Vcap/VA, DmCO, FVC/DLCO and systolic pulmonary arterial pressure (PAP) in the whole study population.

Figure 3 summarizes these correlations. DmCO, Vcap, Vcap/VA, DLCO and FCV/DLCO ratio were highly correlated with systolic PAP in the whole population.

## Discussion

Data on the relevance of partitioning of DLCO in SSc are scarce [18,19]. To our knowledge, our study is the largest assessing combined DLCO/DLNO method in a well defined SSc population clearly separating patients with isolated PAH and no ILD, patients with ILD but no PAH, patients without ILD and PH and patients with both ILD and PH. Moreover presence or absence of ILD was defined by a systematic HRCT and precapillary PH was confirmed in each case by a right heart catheterization. The main result is that partitioning DLCO allowed obtaining either Vcap or Vcap/VA, which were better to detect P(A)H in SSc patients with or without ILD than DLCO alone or ratio like FVC/DLCO.

### Comparison of DmCO and Vcap between patients with isolated PAH and patients with isolated ILD

In the present study, we found that DLCO as well as both Vcap and DmCO were equally reduced in isolated PAH and isolated ILD when compared to SSc patients without these 2 complications. Reduction of both DmCO and Vcap in SSc patients with isolated ILD and isolated PAH has already been described [18,19]. Reduction of both Vcap and DmCO in PAH and ILD SSc patients is probably the consequence of different pathophysiological mechanisms. In PAH, the decrease in capillary flow explains a low Vcap that leads to a reduction in surface area available for gas exchange and therefore a decrease of DmCO [19,28]. DmCO can also be decreased by the thickening of the alveolo-capillary membrane observed in SSc-related PAH. In SSc patients with ILD and no PH, a decrease of DmCO is expected [18] as DmCO is reflecting alteration in the alveolo-capillary membrane through destruction, thickening or infiltration of the membrane as well as the extension of this alteration [28,29]. A reduction of Vcap in ILD may be caused by destruction, obstruction or compression of capillaries in areas of fibrosis [28,29]. Recently, Wémeau-Stervinou et al. also showed that Vcap was also reduced in patients with idiopathic interstitial pneumonia [30] and the explanation was that the capillary density is decreased in these diseases, especially in the most extensively fibrotic region [31]. Moreover, as SSc is a systemic vascular disease, it is highly probable that some degree of pulmonary vascular disease exists in patients with ILD even outside areas of fibrosis. By calculating the Vcap/VA ratio, we tried to focus on capillaries alteration in still well ventilated lung regions participating to gas exchange during the test. We showed that Vcap/VA ratio was significantly lower in isolated PAH than in isolated ILD. This suggests that capillaries are more altered in well ventilated lung in PAH than in ILD in SSc. This Vcap/VA ratio performed much better than other ratio like FVC/DLCO and DLNO/DLCO for the diagnosis of PAH. 

### Role of DmCO and Vcap to suspect PAH in SSc patients without ILD

As guidelines recommend regular PFT and DLCO in SSc patients to detect ILD and PAH, clinicians have often to explore a decrease of DLCO. The main question is whether this decrease in DLCO is reflecting the occurrence of a new ILD or PAH or both. HRCT is a very sensitive tool to diagnose and rule out ILD [26,32]. If a low DLCO is not explained by ILD on HRCT, the next step is to assess the presence of PAH. The role of partitioning DLCO in suspecting PAH in SSc patients has been questioned by Overbeek et al. who showed that DmCO was lower in SSc patients with PAH than in SSc patients without PAH [19]. Conversely there was quite unexpectedly no difference in Vcap between these 2 subgroups [19]. To explain their results, the authors suggested that some patients in the subgroup without PAH could have latent pulmonary vascular disease. However, in this study, some patients with or without PAH had also ILD. Although results were adjusted for interstitial fibrosis, it is likely that in such a complex interaction between parenchymal and vascular disease, the presence of ILD could have interfered with the results. To our knowledge, there are no other available data in the literature. Therefore, we decided to clearly focus on patients without any ILD on HRCT. We confirm that DmCO is decreased in PAH patients but also show that Vcap is reduced in patients with isolated PAH when compared to patients without any lung involvement. In our study, Vcap/VA ratio, DLCO and FVC/DLCO had similar highest performance to detect PAH. 

### Role of DmCO and Vcap to diagnose PH in SSc patients with ILD

Interpretating a low DLCO in the context of ILD in SSc patients is a challenge because DLCO decrease can be ascribed to ILD but also to a possible pulmonary vascular disease. Some studies have suggested that DLCO was lower in SSc patients with ILD and PH than in patients with ILD alone [5]. The FVC/DLCO ratio has also been suggested as a useful index, with a threshold of 1.6 to suspect PAH in ILD patients [14]. To our knowledge, no study has focused on the relevance of partitioning DLCO in this context. In our study, we confirm that DLCO was lower and FVC/DLCO higher in ILD-PH group than in isolated ILD group. Vcap and DmCO were also both reduced in the ILD-PH group. However, Vcap had the highest AUC and sensitivity (83 %) for 100% of specificity at the cut off of 19 mL for the presence of PAH in SSc patients with ILD. Thus, our study shows that partitioning DLCO to obtain Vcap is also better than DLCO alone in the context of ILD to suspect the association between ILD and PH. In our study, the classical FVC/DLCO>1.6 had a very good sensitivity but a poor specificity in this context. 

We deliberately chose to include in the ILD-PH group the 3 patients with a limited ILD extension on HRCT. As we shown, ILD itself, whatever the extension, can influence DLCO and make the interpretation of a low DLCO difficult. Moreover, the clear separation between PH secondary to ILD and the coexistence of PAH and ILD is highly debated in SSc, which is primarily a vascular disease. Moreover, no validated criteria have been published to decide when PH is secondary to ILD in SSc. Therefore, we decided to separate patients with PAH and no ILD at all on one side (PAH group) and patients with precapillary PH and ILD of any extent on the other side (ILD-PH group). Indeed, the first aim of our study was to look if DLCO partition could add some informations for the interpretation of a low DLCO and especially for the presence of precapillary PH, whatever the putative mechanism of this precapillary PH both in patients without ILD and with ILD of any extent.

Our results show that in SSc a low Vcap performs better than a low DLCO to suspect PH in the context of ILD, whatever its extension. However, to ensure the accuracy of our results, we re-ran the analysis after exclusion of the 3 patients with a limited ILD in the ILD-PH group. The results were similar and Vcap remained the parameter with the highest performance to suspect PH in ILD SSc patients. 

### Correlation between DmCO, Vcap, Vcap/VA, DLCO and FVC/DLCO ratio with systolic PAP in the whole population

DmCO, Vcap, Vcap/VA, DLCO and FCV/DLCO ratio were highly correlated with systolic PAP in the whole population. In a recent study, Wemeau-Stervinou et al. also found a correlation between systolic PAP and both Vcap and DmCO in idiopathic interstitial pneumonia and both Vcap and DmCO were altered in these diseases suggesting a common vascular involvement [30].

Our study has some limitations. First, it is retrospective study, which must be validated in prospective studies by a validation cohort and on patients undergoing a systematic right heart catheterization. By its retrospective nature, patients with tricuspid regurgitation velocity was ≤ 2.8 m/s and no additional echocardiographic variables suggestive of PH had no systematic right heart catheterization because we followed the international guidelines and classified them as recommended, as patients with unlikely PH. However, it would be interesting in future prospective studies to perform a systematic right heart catheterization whatever the results of the echocardiography and to correlate hemodynamics with Vcap and Vcap/VA ratio. Second, time interval between HRCT and lung function tests was variable with a median time of 16 months. It is therefore possible that a patient with a normal HRCT develops ILD in the follow-up. However, data in the literature show that it is a rare event [32]. Third, some groups (i.e. PAH) had a low number of patients. However, our results are strengthened by the fact that when pooling patients with a right-heart cathteterization-proven precapillary PH, we found similar results. Moreover, results were still significant after adjusting for age. Finally, partitioning DLCO is not available in routine clinical practice and adds 15 minutes and a forty-seven € cost in France to a standard DLCO test. But our study provides some evidence that this technique is helpful in managing patients with SSc, particularly for the detection of PH in patients with underlying ILD. 

## Conclusion

In SSc, both DLCO, DmCO and Vcap are reduced in SSc patients with ILD and/or precapillary PH. However, Vcap/VA ratio was significantly lower in patients with isolated PAH than in patients with ILD. Moreover, Vcap performed better than DLCO to detect PH in patients with ILD. These results suggest that partitioning of DLCO might be of interest to detect precapillary PH in SSc patients with or without ILD
